# Enhanced recovery after neurosurgery: a narrative review comparing clinical outcomes, protocols, and challenges with standard postoperative care

**DOI:** 10.1097/MS9.0000000000004360

**Published:** 2025-11-18

**Authors:** Tirath Patel, Hamza Yousuf Ibrahim, Rahma Naveed, Aziz Ur Rehman, Maryam Amir, Fatima Nasir, Anum Zia Khan, Nikhilesh Anand

**Affiliations:** a Department of Neurosurgery, Trinity Medical Sciences University School of Medicine, Saint Vincent and the Grenadines; b Jinnah Medical and Dental College, Karachi, Pakistan; c Jinnah Sindh Medical University, Karachi; d Department of Medicine, Akhtar Saeed Medical and Dental College, Lahore, Pakistan; e Department of Medicine, Jinnah Medical and Dental College, Karachi, Sindh, Pakistan; f Department of Medical Education, University of Texas Rio Grande Valley, Edinburg, TX, USA

**Keywords:** Enhanced recovery after surgery (ERAS), healthcare implementation barriers, neurosurgery, perioperative protocols, postoperative outcomes

## Abstract

**Background:**

By integrating evidence-based pre-, intra-, and postoperative therapies, enhanced recovery after surgery (ERAS) protocols have transformed perioperative care in numerous surgical specialties. Although their use in neurosurgery aims to maximize patient recovery, minimize complications, and reduce hospital stays, the evidence remains heterogeneous, and large randomized controlled trials are scarce.

**Objectives:**

This narrative review contrasts the clinical outcomes, advantages, and challenges of ERAS protocols with those of conventional postoperative care in neurosurgery, with a focus on cranial, spinal, pediatric, cancer, and functional procedures.

**Methods:**

A thematic synthesis of peer-reviewed clinical evidence was conducted using PubMed, Scopus, and Google Scholar (2000–2025). Studies comparing ERAS and standard care were reviewed for outcomes including length of stay (LOS), complications, pain control, opioid use, and patient satisfaction. Meta-analyses and systematic reviews were considered for context only, while emphasis was placed on primary clinical data.

**Results:**

ERAS protocols were associated with reduced LOS (3.9 → 3.4 days in spine; 13 → 10 days in cranial surgery), lower postoperative complications [fever −7.9%, unplanned intensive care unit admissions −34.1%], and decreased opioid and patient-controlled analgesia use (−12.1% and −60.2%, respectively). Patient recovery and functional independence improved, although 30-day readmissions slightly increased (+1.0%) before declining by 90 days (−0.3%). Implementation remains inconsistent due to resource limitations, challenges with multidisciplinary coordination, and protocol variability across institutions.

**Conclusion:**

This narrative review demonstrates that ERAS protocols improve recovery, reduce complications, and lower opioid dependence in neurosurgical patients compared with standard care. Despite these advantages, variation in protocol adherence and the limited number of high-quality RCTs restrict universal adoption. Future multicenter trials and standardized frameworks are essential to validate and optimize ERAS integration across neurosurgical practice.

## Introduction

### Background

Enhanced recovery after surgery (ERAS) programs aim to optimize pre-, intra- and postoperative care to improve the quality and speed of recovery in surgical patients. The concept was introduced in 1997 by Danish surgeon Henrik Kehlet. He suggested that while a single technique or drug regimen hasn’t proven to eliminate postoperative morbidity and mortality, multimodal interventions may lead to a significant reduction, and that the risk factors in pre-, intra-, and postoperative surgeries may be addressed through coordinated perioperative protocols. ERAS was formed in 2001 by a group of six surgeons assembled by Professors Ken Fearon, Ollie Ljungqvist, and Professor Kehlet^[1]^. ERAS protocols are multimodal perioperative care pathways designed to achieve early recovery after surgical procedures by maintaining preoperative organ function and reducing the profound stress response following surgery. The key elements of ERAS protocols include preoperative counseling, optimization of nutrition, standardized analgesic and anesthetic regimens, and early mobilization^[2]^. ERAS in neurosurgery is a recent development, and introducing ERAS techniques in craniotomies can significantly influence postoperative care^[3]^.

### Rationale for review

Neurosurgery has substantial risks of complications and death, which can dramatically increase with insufficient postoperative care^[3]^. An ERAS protocol for spinal surgery was developed and first implemented for patients undergoing posterior lumbar decompression and fusion, the most common surgery in our division. The ERAS protocol was found to reduce the risk of adverse events during the perioperative period, promote rehabilitation, and shorten the hospital stay after surgery^[4]^.

Although ERAS has limited adoption due to heterogeneity and a lack of standardization across surgical subspecialities, many institutions are implementing it because it yields better outcomes. Agarwal *et al* stated that few institutions have implemented ERAS protocols in neurosurgery. A survey on the current state of ERAS in neurosurgery was conducted to provide insight into scaling the practice nationally. A total of 39 responses were collected from 38 unique institutions, and 23 reported implementation of neurosurgical ERAS protocols^[5]^. A recent survey of European neurosurgeons found that ERAS protocols were implemented in 36% of healthcare centers, and interestingly, the overall rate of compliance with ERAS protocols was 64.3%^[6]^.

ERAS protocols may be superior to conventional perioperative care in craniotomy patients, with shorter hospital stays, lower postoperative nausea and vomiting (PONV) incidence, and improved postoperative pain scores. Further randomized trials are required to identify the impact of ERAS protocols on the quality of recovery after craniotomy^[7]^. It is important to distinguish between evidence from other surgical fields and evidence specific to the procedures performed and problems encountered in neurosurgery. Thus, future research may focus on unanswered questions regarding perioperative neurosurgery-specific issues in the specific patient groups at risk^[8]^. Given the varying levels of ERAS adoption and the emerging evidence, a narrative review would be essential to provide a comparative analysis between ERAS protocols and standard care in neurosurgery. A systematic review would be early due to variable protocols and the scarcity of neurosurgery-specific RCTs on ERAS, so a narrative review would be more suitable at the moment.

### Objectives

When comparing ERAS with standard postoperative care, Ismail *et al* cover the use of modifications, including how anesthetics function, how to manage pain after surgery, and how to get patients back on their feet. ERAS guidelines place more emphasis on reducing opioid use and encouraging early mobility than more conventional strategies that rely on prolonged bed rest and opioid-based pain management^[7]^.

There are several problems with implementing ERAS into practice in neurosurgery. First, its multidisciplinary nature makes it harder for teams to work together. Also, many parts of ERAS don’t have specific evidence for neurosurgical procedures, which makes some guidelines less useful. Adding too many unnecessary steps to protocols can make them more complicated to use. Another problem is that people often confuse short-term recovery (like a hospital stay) with long-term outcomes (like quality of life after discharge). Pain management is still complex, and strategies that don’t use opioids aren’t always enough for neurosurgical patients. Inconsistent adherence and insufficient long-term data exacerbate challenges to success. Finally, because patients have varying levels of risk, such as frailty and comorbidities, different approaches are needed. There isn’t much research on ERAS interventions in neurosurgery^[8]^.

**HIGHLIGHTS**

- Significant Clinical Improvements: ERAS protocols consistently reduce length of stay, complications, and opioid use while improving functional recovery in cranial, spinal, and pediatric neurosurgery when compared to standard treatment.
- Procedure-Specific Benefits Validated: Despite differences in protocols, strong data support ERAS efficacy in a variety of subspecialties, including reduced PONV in craniotomy, transfusion rates in pediatric scoliosis, and delirium in functional neurosurgery.
- Key Implementation Barriers: Standardization is delayed by interdisciplinary coordination gaps, electronic medical record interoperability challenges, patient non-compliance, and resource limits, leading to delays and workflow interruptions.
- AI Future Opportunities: The integration of telemedicine, remote monitoring, and predictive analytics promises individualized care and early complication detection, but it must address data privacy and digital literacy concerns.
- Critical Research Gaps: Large-scale randomized controlled trials are urgently required for pediatric/geriatric protocols, long-term outcomes, and socioeconomic equity, and should be backed up by legislative changes and funding incentives.

This manuscript is made compliant with the TITAN checklist to ensure transparency in the reporting of artificial intelligence (AI)^[9]^.

## Methodology

This narrative review was carried out to compare standard postoperative care with ERAS protocols in neurosurgery. The review summarizes reported clinical outcomes, explores the practical implications of ERAS, and considers opportunities for standardizing care and addressing barriers to implementation.

### Literature search strategy

A comprehensive search was conducted in PubMed, Scopus, and Google Scholar for studies published between January 2000 and May 2025. The following keywords were used: “Enhanced Recovery After Surgery,” “neurosurgery,” “postoperative care,” “cranial surgery,” “spinal surgery,” “functional neurosurgery,” and “neuro-oncology.”

### Inclusion and exclusion criteria

We included peer-reviewed clinical studies such as randomized trials, observational cohorts, and retrospective analyses that compared ERAS protocols with conventional postoperative care. Case reports, conference abstracts, non-English studies, and papers not directly related to neurosurgical postoperative care were excluded. Meta-analyses and systematic reviews were considered for background information but not as primary evidence.

### Data extraction and synthesis

For each study, details on the design, patient numbers, surgical type, and outcomes, including length of stay, complications, pain management, and recovery, were collected. Instead of statistical pooling, the findings were thematically organized to highlight common patterns and differences between ERAS and standard care. Any differences in interpretation among authors were resolved through discussion.

### Comparative analysis

The review compares ERAS with conventional care across the main neurosurgical fields: cranial, spinal, functional, pediatric, and neuro-oncology/skull base surgery. Outcomes assessed included hospital stay, complication rates, readmission and reoperation rates, pain control, and functional recovery. Whenever possible, emphasis was placed on primary studies to provide a balanced and clinically meaningful overview.

## Overview of ERAS protocols in neurosurgical practice

ERAS protocols are interdisciplinary, evidence-based strategies designed to minimize the physiological stress of surgery and expedite recovery. Initially implemented in colorectal surgery^[10]^, ERAS has gained traction in neurosurgery due to its potential to shorten hospital stays, reduce complications, and optimize resource utilization. In neurosurgical practice, the ERAS pathway consists of five core elements that work synergistically:

### Preoperative optimization

Patients are psychologically and physiologically prepared through targeted interventions, including comorbidity management, nutritional support, and structured education^[11]^. This proactive strategy increases preparedness for postoperative milestones and sets expectations.

### Minimally invasive surgical techniques

Minimally invasive procedures reduce intraoperative tissue damage and blood loss, thereby enhancing postoperative recovery. These techniques have been associated with shorter operative times and quicker mobilization^[12]^.

### Intraoperative anesthesia management

ERAS promotes opioid-sparing anesthesia using multimodal strategies like goal-directed fluid therapy, normothermia maintenance, and dexmedetomidine-based regimens. These interventions reduce the incidence of postoperative cognitive dysfunction and delirium^[13]^.

### Postoperative pain management and early mobilization

Multimodal analgesia, incorporating NSAIDs, acetaminophen, and regional techniques, minimizes opioid use and supports early mobilization within the first 24 hours postsurgery, critical for reducing thromboembolic and pulmonary complications^[12]^.

### Patient education and expectation management

Patients who underwent elective craniotomy within ERAS pathways expressed greater happiness and confidence in their ability to recover. Clear communication about recovery milestones enhances adherence and psychological resilience. Studies show this significantly improves satisfaction and functional outcomes^[14]^.

Compared to conventional postoperative neurosurgical care, which often features inconsistent fasting protocols, delayed ambulation, and reactive pain management, ERAS provides a standardized, proactive framework that enhances both safety and efficiency^[15]^. However, these primary studies show consistent short-term advantages, but they also have drawbacks. The majority of trials are single-center with small sample sizes, and comparability is diminished by institutional protocol variation (e.g., anesthesia regimens, mobilization time). The necessity for multicenter RCTs and standardized pathways is highlighted by the continued underreporting of long-term outcomes, including functional independence and quality of life.

**Figure 1.**
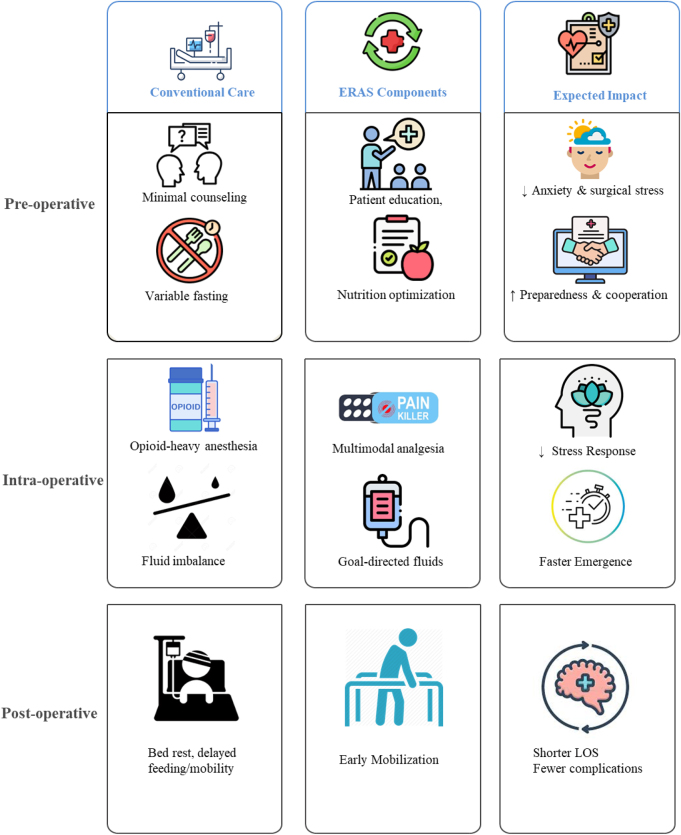
Conceptual framework of ERAS implementation in neurosurgery. ERAS, enhanced recovery after surgery.

Figure 1 provides the conceptual framework illustrating how ERAS modifies perioperative pathways in neurosurgery across pre-, intra-, and postoperative phases, compared with conventional care.

## Procedure-specific applications of ERAS vs. standard care

### Cranial neurosurgery

Several primary studies and meta-analyses^[16–18]^ report the clinical and functional benefits of ERAS in cranial neurosurgery, including reduced surgical stress, shorter hospital and intensive care unit (ICU) stays, and lower rates of postoperative delirium compared with standard care. While meta-analyses provide useful context, most supporting evidence arises from prospective cohorts and small randomized studies. Evidence suggests that patients exhibit resilience with ERAS care, reflecting faster physiological recovery and improved tolerance to perioperative stress. Surgical resilience refers to the time required to return to homeostasis after surgery. Meng *et al* (2024) conducted a meta-analysis focusing on 871 patients across five randomized controlled trials who underwent craniotomy with ERAS care and experienced decreased PONV, a 1.5-day shorter postoperative stay, lower pain scores on postoperative day 1, and no increase in complications^[16]^. The study supported ERAS elements, including early mobilization, fluid de-escalation, multimodal analgesia, and early extubation.

Findings from Stumpo *et al*, a systematic review of 13 studies (primarily prospective cohorts with varying ERAS adherence), showed greater heterogeneity^[19]^, potentially due to inconsistencies in anesthesia, ICU protocols, and mobilization timing. This variation highlights the lack of standardized perioperative care pathways and underscores the need for larger, controlled trials. Aneurysm clipping, while less explored in ERAS literature, implicitly supports broader ERAS benefits. A recent randomized controlled trial from China by Yakar *et al* evaluated 300 elderly patients who underwent aneurysm clipping with an ERAS protocol. These patients had a 1.2-day shorter ICU stay, a 20% faster return to oral feeding, and fewer postoperative complications compared to standard care^[20]^. Although sample sizes remain limited, these data indicate that ERAS can be safely adapted even in high-risk cranial procedures.

### Spine surgery

Leng *et al* (2022) conducted a cohort study involving over 300 patients who underwent anterior cervical spine surgery with an ERAS protocol. Results showed reduced length of stay (LOS), hospital costs, and postoperative complications, along with improved patient satisfaction, without increasing 90-day readmissions or reoperations^[18]^. These outcomes have been consistently reproduced in several prospective series, emphasizing ERAS as a reliable approach for spinal procedures.

A systematic review of 27 studies on lumbar discectomies, spinal fusions, and scoliosis also demonstrated consistent reductions in opioid use, improved pain scores, and shorter hospitalizations^[17]^. However, most studies remain single-center and exhibit heterogeneous protocol adherence, which limits generalizability. A consensus-based analysis by Debono *et al*, encompassing more than 2700 adolescent patients with scoliosis, emphasized the need for integrated psychosocial and nutritional support as part of the ERAS pathway^[20]^. Overall, ERAS in spine surgery offers significant functional benefits, though further randomized trials are needed to strengthen the current evidence.

### Functional neurosurgery

Functional neurosurgery includes procedures like deep brain stimulation (DBS) and epilepsy surgery. Although large RCTs are lacking, observational studies and systematic reviews suggest ERAS care improves outcomes, including reduced opioid use, lower rates of postoperative delirium, and better management of perioperative dopamine disruption in Parkinson’s patients undergoing DBS^[21]^. Current evidence primarily derives from small institutional experiences, indicating the feasibility but not yet the universality of ERAS in these procedures. In epilepsy surgery, ERAS supports safer antiepileptic drug (AED) withdrawal and seizure management^[22]^. Key gaps remain regarding standardized recovery timelines and perioperative medication adjustment protocols, underscoring the need for targeted studies in this domain. Multidisciplinary preoperative assessments are encouraged to identify patients who may benefit from additional rehabilitation, including anxiety management and physical therapy.

### Pediatric neurosurgery

Evidence from craniosynostosis repair^[16,20]^ indicates that ERAS is associated with shorter hospital stays, lower transfusion rates, and improved parental satisfaction. Debono *et al*’s meta-analysis^[20]^ of over 2700 pediatric patients undergoing posterior spinal fusion supports similar outcomes. Nevertheless, variations in perioperative fluid management and analgesia practices continue to affect comparability between centers. Although hydrocephalus surgery is less extensively studied in ERAS literature, structured perioperative protocols show promise. Hagan et al^[22]^ report that ventriculoperitoneal shunt placement in children led to faster return to oral feeding, reduced PONV, and shorter observation periods. These cases also highlight the importance of family education and anticipatory guidance to support early discharge. Despite encouraging findings, pediatric ERAS adoption remains inconsistent, mainly due to the lack of age-specific guidelines and limited high-quality trials.

### Neuro-oncology and skull base surgery

ERAS implementation in neuro-oncology and skull base surgery remains limited due to procedural complexity, long operative durations, and high complication risks. Recent efforts have focused on multidisciplinary protocols involving endocrinology, rehabilitation, pain management, and early mobilization. When appropriately timed, early mobilization reduces ICU stays and promotes functional recovery, though risks such as cerebrospinal fluid (CSF) leaks or vascular instability persist. Primary clinical studies demonstrate feasibility but remain small in scope, reflecting cautious adoption of ERAS in these technically demanding surgeries^**[**^22^**]**^.

In such cases, a patient-specific risk–benefit analysis becomes essential to ensure safe application of ERAS principles. Structured care teams, including neurosurgeons, neuroanesthesiologists, endocrinologists, and physiotherapists, are key to protocol adherence and patient safety. Overall, while the benefits of ERAS are evident across most neurosurgical domains, the limited number of large RCTs and variable adherence patterns highlight the need for standardized, multicenter validation. The outcomes supported by evidence, along with related studies and the limitations of ERAS protocols in various neurosurgical subspecialties, are detailed in Table 1.

**Table 1:** Evidence-based outcomes, supporting studies, and limitations of ERAS protocols across neurosurgical subspecialties

Subspecialty	Key outcomes with ERAS	Supporting studies	Notable limitations
Cranial neurosurgery	Decreased PONV (18% vs. 34%)	^[16,19]^	There was heterogeneity in protocols
	Decreased LOS (4.5 vs. 6 days)		High-quality RCTs were limited.
	Decreased POD1 pain (3.1 vs. 5.4)		
			ERAS adherence was variable among patient groups.
	No increase in complications		
Aneurysm clipping	Decreased ICU stay by 1.2 days	^[20]^	Single RCT
			Focused on the Elderly-only population, hence limited applicability and generalizability
	Faster return to oral feeding by 20%		
	A decrease in the post-op complications		
Spine surgery	Decreased LOS, opioid use, and complications	^[17,18]^	With variable pain scoring and discharge criteria, there was a lack of ERAS standardization in spinal procedures.
	Increase in early ambulation (8–12 hrs vs. 24–36 hrs)		
	No increase in 90-day readmissions		
Pediatric scoliosis	Decrease in transfusion rates (12% vs. 26%)	^[20]^	There is an eventual need for psychosocial and nutritional support
			There are overall, fewer RCTs overall in pediatrics.
	Increase in parental satisfaction (9.2 vs. 7.1)		
Pediatric hydrocephalus	Decreased PONV	^[22]^	Small cohorts were found.
	Decreased time to oral feeding (<12 hrs vs. 24–36 hrs previously)		
			Early-phase implementation is deemed better.
			Caregivers need to be educated on the procedure and the subject.
	Decreased observation time for patients		
Functional neurosurgery	Decreased opioid use, delirium (8% vs. 20%–25%)	^[22]^	The data had mostly observational studies, with no large RCTs found
	Improved dopamine management in Parkinson’s DBS		There was variability in neuropsychiatric comorbidities.
	Better AED tapering in epilepsy surgery		
Neuro-oncology/skull base surgery	Decreased ICU time with early mobilization	^[22]^	CSF leak and instability are some of the listed complications of this surgery
	Potential for faster functional recovery with multidisciplinary protocols		
			In this aspect, there is sparse ERAS-specific outcome data.

AED, antiepileptic drug; CSF, cerebrospinal fluid; DBS, deep brain stimulation; ERAS, enhanced recovery after surgery; ICU, intensive care unit; LOS, length of stay; POD1, postoperative day 1; PONV, postoperative nausea and vomiting; RCT, randomized controlled trial.

## Clinical outcomes: ERAS vs. standard care

ERAS is primarily aimed at improving physiological and corresponding psychological recovery, while reducing inconveniences and the need for secondary interventions. Since the sample sizes for the sources used are far greater than 30, the data may be assumed to be normally distributed, and findings may be confidently assumed to be reliable.

The postoperative LOS is an essential metric for recovery and directly indicates its quality. ERAS has been widely reported to be superior in this regard. A clinical trial (*n* = 129) found that ERAS reduced the median LOS from 13 to 10 days^[23]^. Additionally, the minimum and maximum LOS were also reduced from 5 days and 34 days to 4 days and 29 days, respectively. Another clinical cohort (*n* = 1290) found that median LOS decreased from 3.9 to 3.4 days, confirming previous findings^[24]^. The reduction in LOS also plays an important role in healthcare expenses at the patient’s end and resource utilization at the hospital’s end. While it is cumbersome to calculate improved resource utilization at the hospital’s end, a study shows that ERAS results in up to $386 less spent by the patient on average, based on data from a randomized control trial (*n* = 151)^[17]^.

Another important indicator of recovery is the frequency and magnitude of postoperative complications. ERAS was found to significantly reduce the postoperative fever rate by 7.9%. However, the rate of postoperative seizures was mildly higher by 1.6%^[23]^. Similarly, a study showed that the rate of unplanned postoperative ICU visits was remarkably lower for ERAS by 34.1%^[24]^.

Pain management is another crucial aspect of postoperative recovery. A study showed that ERAS reduced the number of patients requiring overall pain management by 10.3%, and opioid-based pain management by 12.1% – a significant reduction^[23]^. Also, the study provided similar insight, with ERAS exhibiting a mammoth 60.2% reduction in reliance on patient-controlled analgesia. Moreover, long-term pain management (LTPM) was observed to be lower by 31.9% at 1 month and 28.3% at 6 months^[24]^.

Similarly, readmission and reoperation rates provide a measure of effectiveness because 1.0% more ERAS patients were readmitted within 30 days; the trend is contradictory in the longer run, with 0.3% fewer ERAS patients being readmitted within 90 days. Neither of the figures is conclusive^[24]^. A similar randomized control study (*n* = 94) also provides mixed evidence^[25]^. This could be due to the addition of multiple factors during the recovery process after discharge, and patients may practice highly varying levels of care and compliance at home.

Similarly, patient satisfaction is another challenging metric, which is also observed to have inconclusive evidence at this stage. While a study shows that there is not much difference^[24]^, another randomized control study (*n* = 140) shows that ERAS patients had a slightly better overall experience^[26]^. Therefore, more clinical research is needed in this regard. This could be due to highly varying levels of healthcare services expectations, along with the natural variance in staff performance concerning each patient.

The comparative quantitative gains in perioperative metrics between ERAS protocols and conventional care are detailed in Table 2.

**Table 2:** Comparative quantitative gains in perioperative metrics between ERAS protocols and conventional care

Clinical outcome	Metric	ERAS	Standard care	Source
Postoperative LOS	Days	1. Min: 4 days	1. Min: 5 days	^[23]^
		2. Median: 10 days	2. Median: 13 days	
		3. Max: 29 days	3. Max: 34 days	
	Days	Median: 3.4 days	Median: 3.9 days	^[23]^
Postoperative complication rates	%	1. Fever: 14.1%	1. Fever: 21.5%	^[23]^
		2. Seizure: 4.7%	2. Seizure: 3.1%	
	%	ICU visits: 44.9%	ICU visits: 78.9%	^[24]^
Pain management	%	1. Analgesic: 23.1%	1. Analgesic: 33.8%	^[23]^
		2. Weak-opioid: 14.4%	2. Weak-opioid: 12.3%	
		3. Strong-opioid: 4.7%	3. Strong-opioid: 13.8%	
	%	1. PCA: 1.4%	1. Analgesic: 61.6%	^[24]^
		2. LTPM	2. LTPM	
		a. 1-mo: 38.6%	a. 1-mo: 70.5%	
		b. 3-mo: 36.5%	b. 3-mo: 71.9%	
		c. 6-mo: 23.5%	c. simplec. 6-mo: 50.1%	
Avg. hospitalization cost	USD	5880	6266	^[17]^

ERAS, enhanced recovery after surgery; LOS, length of stay; LTPM, long-term pain management; mo, month; PCA, patient-controlled analgesia; USD, United States dollar.

## Summary

### Advantages of ERAS implementation

1. Reduced LOS indicating quicker recovery^[23]^.

2. Efficient utilization of hospital resources and decreased healthcare expenses^[17]^.

3. Reduction in postoperative complications and unplanned ICU visits^[23,24]^.

4. Improved postoperative pain management and reduced reliance on LTPM and opioid use^[24]^.

### Disadvantages of ERAS implementation

1. Increased rate of postoperative re-admission within 30 days^[24]^.

2. Lack of standardized ERAS protocols across all hospitals^[5]^.

3. Inconclusive patient satisfaction scores^[24,26]^.

**Figure 2.**
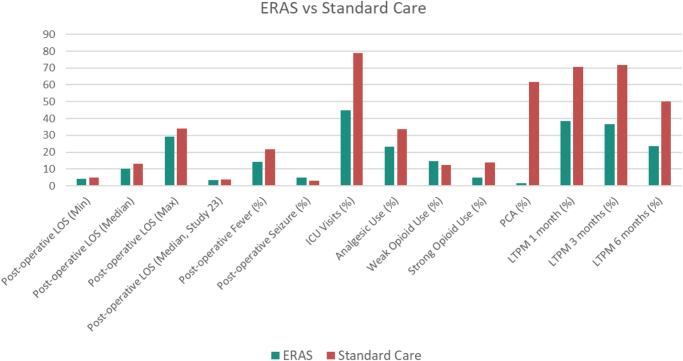
Quantitative summary of ERAS benefits vs. Standard care.

Figure 2 illustrates the Comparative quantitative summary of perioperative outcomes between ERAS and standard postoperative care in neurosurgery, demonstrating consistent reductions in length of stay, postoperative complications, and opioid use across primary studies.

## Barriers and challenges in ERAS implementation

While a pilot implementation on one of Providence Health and Services System’s facilities in the US shows that the adoption of new methodologies results in improved healthcare efficiency^[27]^, another large-scale clinical study on major hospitals in the US (*n* = 38) shows that it also widens the gap between protocol standardization and customization, and is often accompanied by institutional and cultural resistance^[5]^. The study revealed that 52% neurosurgeons reported difficulty in implementing ERAS among the non-surgical staff. They also found it challenging to incorporate ERAS into electronic medical records (EMRs), largely due to a lack of standardization. For example, two major health organizations (Cleveland Clinic and the University of Miami) provide significantly different ERAS protocols for spine surgery, with the former focusing more on reducing perioperative blood transfusions, while the latter emphasizing perioperative counseling, nutrition, and non-opioid analgesics. Moreover, most other neurosurgeons also tailor their protocols based on literature and experience. Therefore, greater effort is needed to facilitate standardization and adoption at both regional and global scales^[5]^.

Additionally, it is imperative to have due regard to comorbidities and preoperative cognitive status. A retrospective cohort study (*n* = 471) finds that patients with adjacent segment disease who underwent highly invasive surgical procedures exhibited significantly better improvement in terms of functional recovery, postoperative deconditioning, and quality of life for both short-term and long-term outcomes^[28]^. Additionally, poor doctor–patient communication and patient non-compliance often hinder the development of ERAS programs. A multicenter study (*n* = 42) showed that doctors also reported that, due to a decrease in postoperative hospital stay, it becomes difficult to follow up with patients for ERAS objectives^[29]^. Moreover, ERAS implementation requires additional workforce and finances, which often don’t come with increased salary or new hirings. This often leads to workplace stress and is counterproductive to original objectives^[29]^.

The key barriers to ERAS implementation, including sources, classifications, and impacts on clinical workflows, are detailed in Table 3.

**Table 3:** Key barriers to ERAS implementation, including sources, classifications, and impacts on clinical workflows

S. No.	Barrier	Source	Category description	Impact on ERAS implementation
1	Difficulty in implementation among non-surgical staff	^[5]^	1. Human resource development	1. Unforeseen delays in implementation timelines
			2. Operations	2. Missed procedures
2	Lack of standardization for EMRs		1. Operations	1. Problems with referrals
			2. Record-keeping	2. Problems with follow-ups
3	Poor doctor–patient communication	^[29]^	1. Operations	1. Unforeseen delays in implementation timelines
				2. Inability to provide patient satisfaction
4	Non-compliance of patients following discharge		2. Recovery and rehabilitation	1. Increased postoperative complications
				2. Increased readmission and reoperation rates

EMRs, electronic medical records; ERAS, enhanced recovery after surgery.

Logically, the relationship between barriers and clinical outcomes is also quite cyclic. For example, difficulty in ERAS implementation among non-surgical staff, lack of EMR standardization, and poor doctor-patient communication collectively result in inefficient recovery, often unnecessarily prolonging LOS. Similarly, non-compliance by patients following discharge directly results in increased postoperative complications and often requires readmission. Almost all scenarios also inflate hospitalization costs.

Therefore, despite its proven benefits, ERAS adoption is generally limited with respect to the standardization, institutional resistance, EMR integration, communication, increased workload, and additional processes. In most cases, the trade-off is not fully justified.

## Future directions and opportunities

While the application of ERAS in neurosurgery continues to expand, several areas require attention to optimize and scale its implementation.

### Standardizing protocols across subspecialties

Variation in practice persists among spine, cranial, and pediatric neurosurgeries. Developing consensus-driven, subspecialty-specific ERAS guidelines is essential for ensuring uniform outcomes and facilitating multicenter comparisons^[13]^.

### Expanding digital health and remote monitoring

Wearable devices, mobile apps, and telehealth platforms enable real-time tracking of pain scores, mobility, and vital signs. These tools can improve adherence to postoperative goals and allow early detection of complications. Integration of such technology into ERAS pathways may shift follow-up care from hospital to home without compromising outcomes^[14]^.

### Leveraging AI

AI-driven risk prediction and outcome modeling could personalize ERAS pathways by tailoring components based on patient profiles. For example, machine learning could help identify patients most likely to benefit from early discharge or flag deviations in recovery trajectories^[30]^.

### Addressing research gaps

There remains a need for high-quality, multicenter trials that validate ERAS across diverse neurosurgical populations. Long-term outcomes, especially in functional recovery and quality of life, are underexplored. Pediatric and geriatric models also require tailored protocols due to unique physiological and psychosocial needs.

### Policy, regulation, and funding

To facilitate broader implementation, regulatory bodies should consider endorsing ERAS protocols within national quality frameworks. Moreover, incentivizing hospitals through bundled payments or quality-based reimbursement could support ERAS adoption, especially in resource-constrained settings.

### Ethical considerations and health equity

As ERAS relies on patient education, access to technology, and coordinated care, disparities in healthcare infrastructure can limit access. Ethically, it is important to ensure that underserved populations are not excluded from the benefits of ERAS. Future research should explore models that adapt protocols to different socioeconomic and cultural contexts.

The strategic future directions for ERAS development, along with the associated advantages and implementation considerations, are presented in Table 4.

**Table 4:** Strategic future directions for ERAS development: advantages and implementation considerations

Future opportunity	Purpose or benefit	Implementation considerations
Standardizing ERAS Guidelines	Reduce variability, improve outcomes^[13]^	Needs a multidisciplinary consensus
Digital health & remote monitoring	Early detection, continuity of care^[14]^	Requires patient digital literacy
AI & predictive analytics	Personalize care, optimize resources^[30]^	Validation and data privacy are needed
Pediatric & geriatric models	Tailored protocols for vulnerable groups^[13]^	Requires new research and resources
Multidisciplinary training	Improve protocol adherence^[11]^	Time and scheduling challenges

AI, artificial intelligence; ERAS, enhanced recovery after surgery.

## Strengths and limitations

This narrative study provides the first comprehensive synthesis of ERAS protocols across neurosurgical subspecialties by combining data from cranial, spinal, pediatric, functional, and oncological surgeries. A multidisciplinary perspective and a focus on translating findings into practical clinical strategies that inform everyday practice are its strengths. The review does have certain limits, though. With a significant emphasis on single-center observational cohorts, the quality of included studies varies, and their narrative design lacks the scientific rigor of a systematic review. Additionally, there is an unequal distribution of evidence across subspecialties, with pediatric and functional neurosurgery showing fewer findings, while spine and cranial surgeries show comparatively better results. These elements must be taken into account when analyzing the findings and suggestions made.

## Conclusion

ERAS is now required; it is a tried-and-true method that, when compared to normal care, speeds up recovery, lowers complications, and restricts the use of opioids during neurosurgery. To date, the data have repeatedly shown significant therapeutic improvements, but the paucity of large-scale, high-quality randomized trials and unequal implementation have hindered progress. In order to proceed, physicians should promptly embrace fundamental ERAS concepts, such as multimodal pain treatment, early mobilization, and standardized perioperative procedures, while adapting them to neurosurgery requirements.

However, in order to achieve wider improvement, coordinated efforts are needed, including interdisciplinary teamwork, institutional and policy-level support, and the integration of digital health tools to guarantee long-term sustainability and adherence. The next question of how efficiently ERAS can be administered to all patients must be the focus of neurosurgical care. Evidence-based and collaboratively driven widespread acceptance could transform post-neurosurgery rehabilitation and establish a new worldwide standard of treatment.

## Data Availability

No new data were generated or analyzed during the study. Data sharing is not applicable to the article, as it is a narrative review.

## Research registration unique identifying number (UIN)

Not required because it is a narrative review.

## Peer and provenance statement

Not commissioned, externally peer-reviewed.
